# Sunitinib-associated hypertension and neutropenia as efficacy biomarkers in metastatic renal cell carcinoma patients

**DOI:** 10.1038/bjc.2015.368

**Published:** 2015-10-22

**Authors:** Frede Donskov, M Dror Michaelson, Igor Puzanov, Mellar P Davis, Georg A Bjarnason, Robert J Motzer, David Goldstein, Xun Lin, Darrel P Cohen, Robin Wiltshire, Brian I Rini

**Affiliations:** 1 Department of Oncology, Aarhus University Hospital, Norrebrogade 44, Aarhus C 8000, Denmark; 2 Massachusetts General Hospital Cancer Center, The Claire and John Bertucci Center for Genitourinary Cancers, 55 Fruit Street, Yawkey 7E, Boston, MA 02114, USA; 3 Division of Hematology–Oncology, Vanderbilt University Medical Center, 2220 Pierce Avenue, 777 Preston Research Building, Nashville, TN 37232-6307, USA; 4 Palliative Medicine and Supportive Oncology Services, Division of Solid Tumor, Cleveland Clinic Taussig Cancer Institute, 9500 Euclid Avenue, Desk R35, Cleveland, OH 44195, USA; 5 Division of Medical Oncology, Sunnybrook Odette Cancer Centre, 2075 Bayview Avenue, Toronto, ON M4N 3M5, Canada; 6 Department of Medicine, Genitourinary Oncology Service, Memorial Sloan-Kettering Cancer Center, 1275 York Avenue, New York, NY 10021, USA; 7 Department of Medical Oncology, Prince of Wales Hospital, Randwick, NSW 2031, Australia; 8 Pfizer Oncology, 10646 Science Center Drive, La Jolla, CA 92121, USA; 9 Pfizer Oncology, Walton Oaks, Dorking Road, Walton-on-the-Hill, Tadworth, Surrey KT20 7NS, UK; 10 Cleveland Clinic Taussig Cancer Institute, Glickman Urological Institute, 9500 Euclid Avenue, Desk R35, Cleveland, OH 44195, USA

**Keywords:** sunitinib, metastatic renal cell carcinoma, biomarkers, adverse events, multivariate, neutropenia, hypertension

## Abstract

**Background:**

Metastatic renal cell carcinoma (mRCC) prognostic models may be improved by incorporating treatment-induced toxicities.

**Methods:**

In sunitinib-treated mRCC patients (*N*=770), baseline prognostic factors and treatment-induced toxicities (hypertension (systolic blood pressure ⩾140 mm Hg), neutropenia (grade ⩾2), thrombocytopenia (grade ⩾2), hand–foot syndrome (grade >0), and asthenia/fatigue (grade >0)) were analysed in multivariate analyses of progression-free survival (PFS) and overall survival (OS) end points.

**Results:**

On-treatment neutropenia and hypertension were associated with longer PFS (*P*=0.0276 and *P*<0.0001, respectively) and OS (*P*=0.0014 and *P*<0.0001, respectively), independent of baseline prognostic factors, including International Metastatic Renal Cell Carcinoma Database Consortium (IMDC) criteria. By 12-week landmark analysis, neutropenia was significantly associated with longer PFS and OS (*P*=0.013 and *P*=0.0122, respectively) and hypertension or hand–foot syndrome with longer OS (*P*=0.0036 and *P*=0.0218, respectively). The concordance index was 0.65 (95% CI: 0.63−0.67) for IMDC classification alone and 0.72 (95% CI: 0.70−0.74) when combined with hypertension and neutropenia. Considering hypertension and neutropenia (developing both *vs* neither) changed IMDC-predicted median OS in each IMDC risk group (favourable: 45.3 *vs* 19.5 months; intermediate: 32.5 *vs* 8.0 months; poor: 21.1 *vs* 4.8 months).

**Conclusions:**

On-treatment neutropenia and hypertension are independent biomarkers of sunitinib efficacy and may add prognostic accuracy to the IMDC model.

The rapid development and approval of several molecularly targeted agents for the treatment of advanced renal cell carcinoma (RCC) has resulted in significant clinical benefit to patients by providing multiple treatment options. The identification of baseline prognostic criteria has enabled classification of patients into favourable, intermediate, and poor risk groups, providing guidance regarding selection and sequencing of therapy (Motzer *et al*, 2002; Heng *et al*, 2009; Ljungberg *et al*, 2010; Patil *et al*, 2011; Motzer *et al*, 2011; Heng *et al*, 2013). However, baseline risk categories are dynamic and can change during treatment (Ko *et al*, 2015). Therefore, investigation into on-treatment efficacy biomarkers in order to adjust prognosis after starting therapy is warranted.

Several potential serum-, radiological-, and tissue-based biomarkers have been investigated across multiple agents, including circulating soluble proteins associated with angiogenic pathways (e.g., vascular endothelial growth factor (VEGF)), functional imaging, VEGF single-nucleotide polymorphisms, and the tumour marker hypoxia-inducible factor 1-alpha (Schneider *et al*, 2008; Figlin *et al*, 2009; Peña *et al*, 2010; Escudier *et al*, 2011; Garcia-Donas *et al*, 2011; Xu *et al*, 2011; Muriel López *et al*, 2012; Tran *et al*, 2012; Zurita *et al*, 2012; Bex *et al*, 2014; Harmon *et al*, 2014; Mains *et al*, 2014). However, none of these biomarkers have been integrated into a prognostic model or validated for use in RCC (Jain *et al*, 2009). Moreover, given the investment of patient resources, time, cost, and expertise associated with each, none represent an ideal biomarker for clinical use. An alternative to such biomarkers are mechanism-based adverse events (AEs) that reflect ‘on-target' effects of a molecularly targeted agent and its inhibition of a given pathway (Dienstmann *et al*, 2011). Such on-target AEs may be used as surrogates of pharmacokinetic and pharmacodynamic effect, as well as potential predictors of efficacy.

Sunitinib malate (SUTENT; Pfizer Inc., New York, NY, USA) is an oral multitargeted inhibitor of VEGF receptors, platelet-derived growth factor receptors, and other receptor tyrosine kinases that is approved internationally for the treatment of advanced RCC (Sunitinib malate (SUTENT) prescribing information, 2014). Prior retrospective analyses using a pooled database of five prospective sunitinib clinical trials in patients with metastatic RCC (mRCC) identified treatment-associated hypertension as a biomarker of efficacy (Rini *et al*, 2011). A four-fold improved survival rate was seen in patients who develop hypertension during therapy compared with patients without hypertension.

In the present analyses, we used the same pooled database to separately evaluate hand–foot syndrome, asthenia and/or fatigue, neutropenia, and thrombocytopenia as potential biomarkers of sunitinib efficacy in individual AE models. These AEs were chosen because they are common, manageable, readily, and systematically measurable, and potentially associated with the intended target inhibition of sunitinib. The relative strength and independence of each biomarker, including hypertension, were then assessed in the final combined multivariate analyses. In addition, independent biomarkers were incorporated into our own prognostic model as well as the International Metastatic Renal Cell Carcinoma Database Consortium (IMDC) model (Heng *et al*, 2013).

## Materials and methods

### Patients

Pooled retrospective analyses included patients from five prospective clinical trials (Motzer *et al*, 2006a, 2006b; Escudier *et al*, 2009; Motzer *et al*, 2009; Barrios *et al*, 2012). Common eligibility criteria included age ⩾18 years with histologically confirmed mRCC, presence of measurable disease, no known brain metastases, Eastern Cooperative Oncology Group (ECOG) performance status 0/1, and adequate organ function. All patients gave written informed consent.

### Study designs and assessments

The analyses included pooled data from 770 mRCC patients who received sunitinib in both the first-line (*n*=494; 64%) and cytokine-refractory (*n*=276; 36%) treatment settings (Motzer *et al*, 2006a, 2006b; Escudier *et al*, 2009; Motzer *et al*, 2009; Barrios *et al*, 2012). Sunitinib was administered orally at either a starting dose of 50 mg once daily on a 4-weeks-on–2-weeks-off schedule (Schedule 4/2), in repeated 6-week cycles (*n*=544; 71%), or 37.5 mg continuously once daily (*n*=226; 29%). Treatment continued until disease progression, lack of clinical benefit, unacceptable toxicity, or consent withdrawal.

Efficacy end points included objective response (OR) and progression-free survival (PFS), assessed by investigators using Response Evaluation Criteria in Solid Tumours (Therasse *et al*, 2000) and overall survival (OS). The AEs neutropenia, thrombocytopenia, hand–foot syndrome, and asthenia/fatigue were recorded regularly and graded according to the National Cancer Institute Common Terminology Criteria for Adverse Events (CTCAE), version 3.0, with severity grades for neutropenia and thrombocytopenia based on absolute neutrophil and platelet counts, respectively. The frequency of haematology assessments varied across the studies; however, in general, most patients were assessed at screening within 1–3 weeks prior to study entry, predose on Cycle 1 Day 1, at least every 2 weeks during the first 6-week cycle, and every 4 weeks thereafter until end of treatment or withdrawal. As previously studied (Rini *et al*, 2011), hypertension was defined by maximum systolic blood pressure (SBP) of ⩾140 mm Hg, measured in the clinic on Days 1 and 28 of each 6-week treatment cycle. Results of analyses using hypertension defined by diastolic blood pressure ⩾90 mm Hg were similar (Rini *et al*, 2011).

The studies were run in accordance with the International Conference on Harmonization Good Clinical Practice guidelines (or the Declaration of Helsinki) and applicable local regulatory requirements and laws and approved by the institutional review boards or independent ethics committees of each participating centre.

### Analytical and statistical methods (individual AE models)

In the individual AE models, PFS and OS were estimated by Kaplan–Meier method, and a log-rank test was used to compare results between groups of patients with *vs* without the AE in question: neutropenia (grade ⩾2), thrombocytopenia (grade ⩾2), hand–foot syndrome (grade >0), or asthenia/fatigue (grade >0). The grade ⩾2 threshold was chosen for neutropenia and thrombocytopenia based on prior chemotherapy studies in which this severity level for each AE was associated with improved prognosis and, historically, formed the basis for a dose individualisation approach using ‘toxicity-adjusting dosing' (Gurney, 2002). A grade >0 threshold was chosen for hand−foot syndrome and asthenia/fatigue because of the small numbers of patients with higher severity grades for these AEs, limiting the statistical power of using a higher threshold.

The influence on PFS and OS of several prognostic factors, including sunitinib relative dose intensity (the ratio of actually received to intended sunitinib dose) for the overall treatment period and previously identified prognostic factors (including the Memorial Sloan-Kettering Cancer Center (MSKCC) prognostic criteria; Motzer *et al*, 2002) were analysed using a separate multivariate Cox proportional hazards model for each AE; factors with a *P*-value <0.2 in univariate analysis (based on two-sided Wald chi-squared tests) were included in the multivariate model to identify if the AE in question was a significant independent predictor (*P*<0.05).

In addition, a Cox proportional hazards model with each AE as a time-dependent covariate (to address potential bias from longer drug exposure) was used to further estimate hazard ratios for PFS and OS.

Finally, separate landmark analyses using the Kaplan–Meier method were performed at 6 and 12 weeks for each AE (in which patients were grouped based on the occurrence of the AE prior to these time points) to assess the correlations between early AE occurrence and outcome. Patients who died or had disease progression before the landmark time point were excluded from the OS and PFS analyses, respectively.

### Analytical and statistical methods (combined AE models)

In the final combined AE analyses, a multivariate Cox proportional hazards regression model was used to simultaneously analyse the independent predictive value of all the preceding AE biomarkers, including hypertension (Rini *et al*, 2011) and other prognostic factors, on clinical outcome. Thus the following were used as covariates for association with OR, PFS, and OS: treatment-induced hypertension (SBP⩾140 mm Hg); neutropenia (grade⩾2); thrombocytopenia (grade⩾2); hand–foot syndrome (grade >0); asthenia/fatigue (grade >0); sunitinib dose reduction; sunitinib relative dose intensity for the overall treatment period; and previously identified prognostic factors, including the MSKCC criteria (Motzer *et al*, 2002) and prognostic criteria developed for mRCC patients receiving VEGF inhibitors (as integrated into the IMDC model; Heng *et al*, 2013). (Note: pretreatment hypertension was not included as a covariate, but uncontrolled hypertension was an exclusion criterion in all clinical trials (Pfizer, data on file; Rini *et al*, 2011). Each factor was investigated in univariate and then multivariate analyses using a Cox proportional hazards model; *P*-values were based on two-sided Wald chi-squared tests.

The multivariate analysis was repeated for PFS and OS using a 12-week landmark (i.e., AEs evaluated up to the first 12 weeks of treatment) to address potential bias from misclassification of patients who may not have remained on study long enough for an AE to be observed. Patients who died or had disease progression before the landmark time point were excluded from the OS and PFS analyses, respectively.

The ability of the AEs hypertension and neutropenia to improve the prognostic accuracy of the IMDC model was evaluated using the concordance index (C-index), which was calculated using individual IMDC scores followed by the addition of hypertension and neutropenia. A C-index of 0.5 represents no predictive discrimination, and a C-index of 1 represents perfect ability to distinguish patients.

To control for the prognostic impact of the baseline status of neutrophil counts, subgroup analyses were performed to evaluate the impact of eight different clinical scenarios (‘8-group' analysis) on efficacy outcomes (OR, PFS, and OS). The subgroups were based on combined baseline neutrophil count and nadir neutropenia grade and hypertension status during treatment. PFS and OS were estimated by Kaplan–Meier method, with corresponding 95% confidence intervals (CIs) calculated. The prognostic impact of adding on-treatment hypertension and neutropenia to baseline IMDC classification was evaluated using OS alone.

All combined AE analyses were performed separately for patients on Schedule 4/2 and on any dose/schedule (Schedule 4/2 and continuous daily dosing combined).

## Results

### Patients

The pooled database comprised 770 patients included in five clinical trials. Most patients in these retrospective analyses were male, with median ages ranging from 56 to 62 years in the trials comprising the pooled database (Table 1). More than 98% had a diagnosis of RCC with clear cell histology, and most had an ECOG performance status of 0/1 and had undergone prior nephrectomy.

### Incidences of AEs during treatment

Among the 770 patients included in the analyses, 614 (80%), 365 (47%), 172 (22%), 179 (23%), and 583 (76%) experienced on-treatment SBP-defined hypertension, neutropenia (grade ⩾2), thrombocytopenia (grade ⩾2), hand–foot syndrome (grade >0), and asthenia/fatigue (grade >0), respectively. Hypertension was more frequent than in previous studies because it was defined here by SBP rather than by CTCAE (Sunitinib malate (SUTENT) prescribing information, 2014).

### Individual AE models of associations between AEs and efficacy

Hand–foot syndrome, asthenia/fatigue, neutropenia, and thrombocytopenia were all statistically significantly associated with PFS and OS in univariate analyses (Table 2, which includes previously reported data for hypertension). In separate multivariate analyses for each AE, in which established baseline prognostic factors were included and other AEs were omitted, hand–foot syndrome, asthenia/fatigue, neutropenia, and thrombocytopenia were all statistically significantly associated with PFS and OS (Table 2). In addition, both neutropenia and hypertension remained statistically significantly associated with both PFS and OS by time-dependent covariate analysis (Table 2). By separate 12-week landmark analyses, neutropenia and hypertension were significantly associated with both PFS and OS, and hand–foot syndrome was significantly associated with OS; asthenia/fatigue and thrombocytopenia were not significantly associated with either PFS or OS (Table 2).

### Combined AE (final multivariate) models of associations between AEs and efficacy

The final multivariate analyses included hand–foot syndrome, asthenia/fatigue, neutropenia, thrombocytopenia, and hypertension (as previously reported), as well as established baseline prognostic factors, including individual factors integrated in the MSKCC and IMDC prognostic models. Development of any of the five AEs at any time point during sunitinib treatment, regardless of dose/schedule, was statistically significantly associated with greater OR (Table 3, which reports the results for AEs only), independent of baseline prognostic factors (Supplementary Table S1, which includes the full model with results for all covariates). In addition, the occurrence of neutropenia and hypertension during treatment was significantly associated with both longer PFS and OS (Table 4, which reports the results for AEs only), independent of baseline prognostic factors (Supplementary Table S2, which includes the full model with results for all covariates); however, the occurrence of asthenia/fatigue at any time was significantly associated with longer PFS only, and the occurrence of hand–foot syndrome at any time was significantly associated with longer OS only.

By 12-week landmark analyses (Table 4), neutropenia was significantly associated with both longer PFS and OS, the occurrence of hypertension or hand–foot syndrome was significantly associated with longer OS, and asthenia/fatigue results were not significant in any of the landmark analyses. Results were similar regardless of treatment schedule. In addition, the AEs significantly associated with outcome did so independently of baseline prognostic factors (Supplementary Table S2).

The occurrence of thrombocytopenia was not significantly associated with longer PFS or OS in any of the analyses (at any time point or in the 12-week landmark analyses), regardless of treatment schedule (Table 4).

The C-index for IMDC classification alone was 0.65 (95%: CI 0.63−0.67), increasing to 0.72 (95% CI: 0.70−0.74) for IMDC combined with hypertension and neutropenia.

Dose reduction for any reason, which was also included as a covariate in the multivariate analysis models, was significantly associated with improved efficacy outcomes (OR, PFS, and OS) in the analyses of AEs occurring at any time point and regardless of treatment schedule (Supplementary Tables S1 and S2); however, it was not significantly associated with survival outcomes (PFS and OS) in the 12-week landmark analyses (Supplementary Table S2).

According to change-from-baseline (Cox proportional hazards) analyses in which asthenia/fatigue and hand–foot syndrome were excluded (data not shown), the following factors were significantly associated with PFS: (1) ⩾10 (or ⩾15) mm Hg change in SBP or a change in SBP ⩾ median (of the maximum change from baseline) and (2) a change in neutrophil counts ⩾ median (of the worst change from baseline). The change-from-baseline analyses found that the same changes in SBP were also significantly associated with OS and that a change in platelet counts ⩾ median (of the worst change from baseline) was significantly associated with OS as well. Although these analyses did not duplicate the findings of the combined multivariate analyses (owing to the lack of association between changes in neutrophil counts and OS), they were generally supportive.

### Incorporating biomarkers into a prognostic model

**Figure 1:**
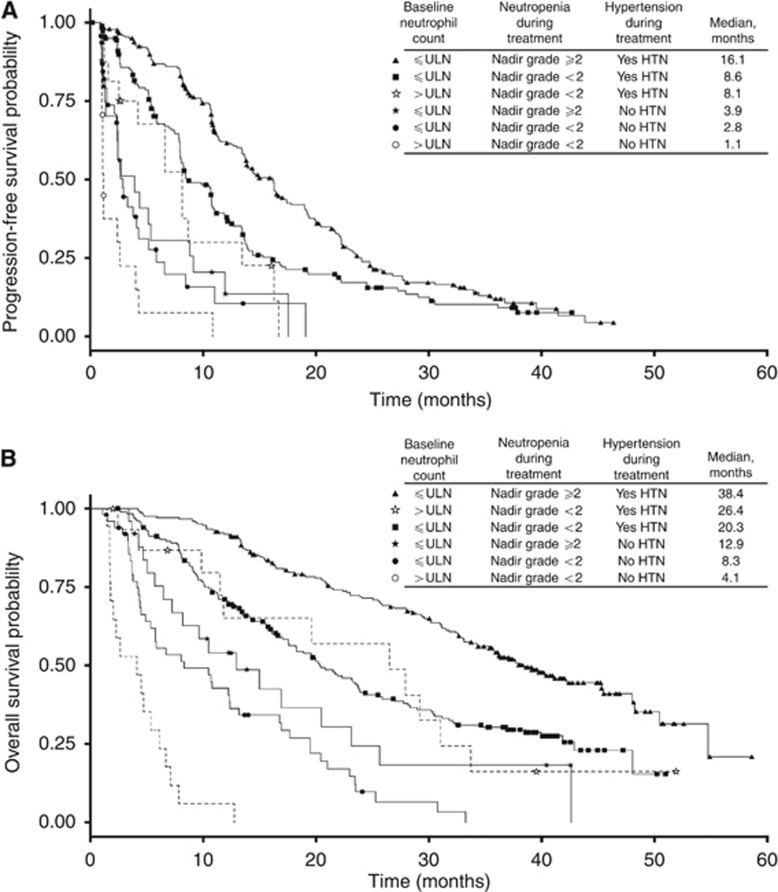
**Prognostic model based on baseline neutrophil status (normal *vs* elevated) with the addition of on-treatment status of hypertension (HTN; yes *vs* no) and neutropenia (yes *vs* no) (‘8-group' analysis).** All patients were treated with sunitinib on Schedule 4/2. (**A**) PFS; (**B**) OS. ULN=upper limit of normal.

We examined the impact of on-treatment hypertension and neutropenia in combination with established baseline risk factors. First, we used a simple model based on baseline neutrophil status, that is, elevated *vs* normal (⩽ upper limit of normal; Figure 1 and Supplementary Table S3). Patients on Schedule 4/2 with elevated baseline neutrophil counts who did not subsequently experience neutropenia or hypertension during therapy had an OR rate (ORR) of 0%, median PFS of 1.1 months, and median OS of 4.1 months. Almost no patients with elevated baseline neutrophils, regardless of dose/schedule, experienced neutropenia. However, if patients on Schedule 4/2 with elevated baseline neutrophils subsequently experienced hypertension during therapy, they had an ORR of 44%, median PFS of 8.1 months, and median OS of 26.4 months. Patients with normal neutrophil counts at baseline who subsequently experienced both on-treatment neutropenia and hypertension had an ORR of 65%, median PFS of 16.1 months, and median OS of 38.4 months.

**Figure 2:**
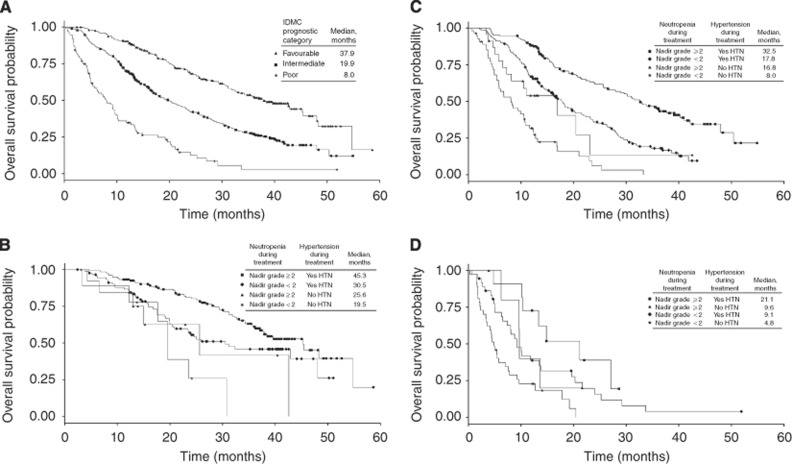
**Incorporating on-treatment neutropenia and hypertension into the IMDC model leads to improved prognostic accuracy.** Prognostic model based on (**A**) baseline IMDC status, with the addition of on-treatment status of neutropenia and hypertension in patients with (**B**) IMDC favourable prognosis, (**C**) IMDC intermediate prognosis and (**D**) IMDC poor prognosis.

We next used the IMDC prognostic model. For patients categorised in the IMDC favourable-, intermediate-, and poor-risk groups, the median OS was 37.9, 19.9, and 8.0 months, respectively (Figure 2A). Patients in the favourable-risk group were then analysed by adding on-treatment hypertension and neutropenia. For patients in the favourable-risk group with both hypertension and neutropenia compared with no hypertension and neutropenia, median OS more than doubled (45.3 *vs* 19.5 months) (Figure 2B). For patients in the intermediate-risk group with both hypertension and neutropenia compared with no hypertension and no neutropenia, median OS was four-fold longer (32.5 *vs* 8.0 months) (Figure 2C). For patients in the poor-risk group who developed both hypertension and neutropenia compared with no hypertension and neutropenia, median OS was also four-fold longer (21.1 *vs* 4.8 months) (Figure 2D).

## Discussion

The present study is the largest and the most comprehensive assessment of known prognostic factors and multiple on-treatment toxicities in patients with mRCC. We demonstrated that on-treatment development of neutropenia and hypertension and, to a lesser degree, hand–foot syndrome, are independent biomarkers of sunitinib efficacy. Development of neutropenia or hypertension or, to a lesser degree, hand–foot syndrome, on treatment predicted improved outcomes, and development of both neutropenia and hypertension predicted even better outcomes. Moreover, incorporating on-treatment neutropenia and hypertension into the IMDC model appeared to add prognostic accuracy.

Recently, Ko *et al* (2015) demonstrated that baseline-risk categories are dynamic and change during treatment. In patients whose prognosis was evaluated using IMDC criteria prior to both first- and second-line therapy, approximately 40% had changes in their initial prognostic assessment: from poor to intermediate, favourable to intermediate, or intermediate to poor prognosis. Thus baseline-risk allocations into prognostic categories are not static but are dynamic variables that need readjustment during therapy. Our finding that addition of on-treatment hypertension and neutropenia to baseline IMDC prognostic information predicted a four-fold survival difference within both the IMDC intermediate- and poor-risk groups, and a two-fold survival difference in the favourable-risk group points to hypertension and neutropenia as simple means of adjusting prognosis during first-line therapy. Accurate reassessment of prognosis during therapy may translate group-level prognostic information into individual patient-level information, thereby improving prognostication during treatment. Moreover, failure to develop neutropenia and/or hypertension on treatment could be included as a stratification factor in prospective studies assessing whether dose adjustment or switching to an alternative treatment strategy may change clinical outcome.

The results of these analyses are consistent with the known mechanisms by which sunitinib induces these AEs and how these mechanisms relate to disease progression. With regard to myelosuppression, because stem cell factor receptor and FMS-like tyrosine kinase 3, as well as VEGF receptors, are involved in hematopoietic cell proliferation and differentiation, their inhibition by sunitinib may cause myelosuppression through on-target mechanisms (Kumar *et al*, 2009). This explains why neutropenia, albeit mostly grade 1/2 in severity, is frequently associated with sunitinib treatment (Sunitinib malate (SUTENT) prescribing information, 2014). In addition, several studies have demonstrated that tumours stimulate neutrophils to promote angiogenesis (e.g., via release of neutrophil-derived VEGF) and immunosuppression, as well as migration, invasion, and metastasis; that may explain the poor prognosis of patients with pretreatment neutrophilia (Donskov, 2013), which may or may not be overcome by therapy (Taichman *et al*, 1997; Jensen *et al*, 2009; Carus *et al*, 2013; Dumitru *et al*, 2013). However, this may also explain why sunitinib-induced neutropenia is associated with clinical benefit. With regard to hypertension, VEGF inhibition has been shown to decrease nitric oxide production, leading to vasculature constriction and a reduction in sodium ion renal excretion, resulting in hypertension (Hood *et al*, 1998; Van Heeckeren *et al*, 2007), with evidence of the involvement of other renovascular mechanisms as well (Dechend and Luft, 2008). As previously hypothesised (Rini *et al*, 2011), the susceptibility of blood vessels to VEGF blockade, resulting in hypertension, may also be linked to the susceptibility of tumour vessels to VEGF blockade, providing a biological underpinning for the biomarker results. Of note, however, the prior hypertension analyses also found that baseline antihypertensive medication use was significantly associated with longer OS and that the addition of antihypertensive medication during sunitinib treatment did not negatively impact improved survival for hypertensive patients (Rini *et al*, 2011). These findings suggested the possibility of inherent host biology independent of VEGF inhibition that predisposes patients to a favourable outcome. Such a hypothesis would be compatible with our findings, in which hypertension was significantly associated with OS, but not PFS, in the 12-week landmark analyses and also explain the apparent additive interaction between on-treatment hypertension and neutropenia associated with prolonged survival in the prognostic model analysis. Dose reduction was also not significantly associated with survival outcomes in the 12-week landmark analyses, supporting the hypothesis that hypertension, neutropenia, and hand–foot syndrome are a reflection of underlying tumour biology rather than simply a reflection of higher sunitinib exposure. Further research into underlying biological mechanisms and methods for using these biomarkers to optimise therapy for individual patients is warranted.

Our results obtained in patients participating in clinical trials are consistent with recent data from non-trial patients with mRCC treated with sunitinib at a single institution (Rautiola *et al*, 2014) and with recent data from a complete national cohort of patients with mRCC treated with first-line tyrosine kinase inhibitors or interleukin-2-based immunotherapy, assessed within the first 12 weeks of treatment (Soerensen *et al*, in press). Taken together, on-treatment hypertension and neutropenia may therefore be considered to be new, independent, validated prognostic factors. Our study is the first to demonstrate a clear adjustment in the prognosis of all IMDC risk subgroups by adding on-treatment hypertension and neutropenia to baseline IMDC risk classification.

These analyses employed a large database, providing a robust data set. However, in addition to the usual issues associated with retrospective analyses (e.g., lack of internal validation), the following limitations may have confounded results: variability in AE assessment among the studies (e.g., particularly for subjective AEs such as asthenia/fatigue), including lack of central laboratory assessment of neutrophil and platelet counts and variation in their assessment frequency across studies; and lack of pharmacokinetic data coinciding with the occurrence of each AE biomarker, precluding investigation of the potential impact of drug exposure (although the fact that not all AEs evaluated were identified as independent biomarkers decreases the likelihood of such an epiphenomenon). Also, there may be other potential AEs that should have been included in this analysis, such as sunitinib-induced hypothyroidism, which has been reported as a potential biomarker (Schmidinger *et al*, 2011) (routine monitoring of thyroid function tests was not standard within the early clinical studies of sunitinib used in our analyses, and therefore these data are incomplete, precluding their inclusion). In addition, the clinical utility of a 12-week landmark for biomarker analysis rather than an earlier time point may be limited.

In conclusion, the analyses reported herein identify on-treatment neutropenia and hypertension as prognostic factors. Moreover, incorporating hypertension and neutropenia into the IMDC model leads to improved prognostic accuracy. Thus identification of neutropenia and hypertension and, to a lesser degree, hand–foot syndrome, during sunitinib therapy may enable physicians to predict which patients are mostly likely to benefit from therapy. Moreover, lack of neutropenia and/or hypertension within 12 weeks could be incorporated as a stratification factor in prospective studies assessing whether dose adjustment or switching to an alternative treatment strategy may change clinical outcome. In the meantime, providers who observe these AEs in their patients should be encouraged to continue sunitinib therapy, while managing AEs with standard medical treatment, with or without dosing interruption and/or dose reduction, as clinically indicated.

## Figures and Tables

**Table 1:** Baseline patient characteristics

Characteristic	Second-line, Schedule 4/2 phase II trial (Motzer *et al*, 2006a) (*n*=63)	Second-line, Schedule 4/2 phase II trial (Motzer *et al*, 2006b) (*n*=106)	First-line, Schedule 4/2 phase III trial (Motzer *et al*, 2009) (*n*=375)^a^	First-line, Schedule CDD phase II trial (Barrios *et al*, 2012) (*n*=119)	Second-line, Schedule CDD phase II trial (Escudier *et al*, 2009) (*n*=107)
Median (range) age, years	60 (24–87)	56 (32–79)	62 (27–87)	58^b^ (24–78)	59 (29–80)
Male/female, *n* (%)	68/32	63/37	71/29	76/24	82/18
**ECOG PS, *n* (%)**					
0	34 (54)	58 (55)	231 (62)	63 (53)	61 (57)
1	29 (46)	48 (45)	144 (38)	56 (47)	45 (42)
⩾2	0	0	0	0	1 (1)
**Histology, *n* (%)**					
Clear cell	55 (87)	105 (99)	375 (100)	119 (100)	104 (97)
Other	8 (13)	1 (1)	0	0	3 (3)
Prior nephrectomy, *n* (%)	58 (92)	106 (100)	340 (91)	112 (94)	100 (93)
Prior cytokine therapy, *n* (%)	63 (100)	106 (100)	0	0	107 (100)
Prior radiation therapy, *n* (%)	25 (40)	20 (19)	53 (14)	15 (13)	NA
**No. of disease sites, *n* (%)**					
1	8 (13)	13 (12)	55 (15)	30 (25)	12 (11)
⩾2	55 (87)	93 (88)	320 (85)	87 (73)^c^	95 (89)

Abbreviations: CDD=continuous daily dosing; ECOG PS=Eastern Cooperative Oncology Group performance status; NA=not available.

a The 375 patients cited in the table are those who received sunitinib in this trial.

b Mean value presented.

c Data missing for two patients.

**Table 2:** Summary of individual AE models for the associations between adverse events and survival end points for mRCC patients receiving sunitinib

						6-week landmark			12-week landmark		
Efficacy end point	Median time to progression/survival event, months		*P*-value	Multivariate analysis,^a^ HR (*P*-value)	Time-dependent covariate analysis, HR (*P*-value)	Median time to progression/survival event, months (*n*)		*P*-value	Median time to progression/survival event, months (*n*)		*P*-value
**Hypertension during treatment (Rini *et al*, 2011)**											
	SBP⩾140 mm Hg (*n*=442)	SBP <140 mm Hg (*n*=92)		SBP⩾/<140 mm Hg	SBP⩾/<140 mm Hg	SBP⩾140 mm Hg	SBP <140 mm Hg		SBP⩾140 mm Hg	SBP <140 mm Hg	
PFS	12.5	2.5	<0.001	0.241 (<0.001)	0.603 (<0.001)	13.4	10.8	0.031	13.6	10.8	0.015
OS	30.9	7.2	<0.001	0.284 (<0.001)	0.332 (<0.001)	32.2	20.3	<0.001	31.1	18.2	<0.001
**Hand–foot syndrome during treatment**											
	Yes (*n*=179)	No (*n*=591)		Yes/No	Yes/No	Yes	No		Yes	No	
PFS	14.3	8.3	<0.0001	0.707 (0.001)	0.874 (0.211)	12.3	9.4	0.251	10.6	9.0	0.535
OS	38.2	18.9	<0.0001	0.519 (<0.001)	0.539 (<0.001)	43.8	21.6	0.007	37.4	20.3	<0.001
**Asthenia/fatigue during treatment**											
	Yes (*n*=583)	No (*n*=187)		Yes/no	Yes/no	Yes	No		Yes	No	
PFS	10.9	6.4	<0.001	0.527 (<0.001)	0.941 (0.543)	9.6	9.3	0.197	9.2	8.4	0.821
OS	26.2	15.0	<0.001	0.634 (<0.001)	0.890 (0.296)	24.2	19.7	0.128	23.8	18.9	0.086
**Neutropenia during treatment**											
	Gr ⩾2 (*n*=366)	Gr <2 (*n*=404)		Gr ⩾/<2	Gr ⩾/<2	Gr ⩾2	Gr <2		Gr ⩾2	Gr <2	
PFS	13.6	7.1	<0.0001	0.520 (<0.0001)	0.759 (0.0032)	9.7	9.4	0.615	11.0	8.0	0.005
OS	35.6	15.8	<0.0001	0.415 (<0.0001)	0.467 (<0.0001)	26.5	21.9	0.041	29.7	19.0	0.001
**Thrombocytopenia during treatment**											
	Gr ⩾2 (*n*=101)	Gr <2 (*n*=669)		Gr ⩾/<2	Gr ⩾/<2	Gr ⩾2	Gr <2		Gr ⩾2	Gr <2	
PFS	13.7	8.8	0.001	0.658 (0.003)	0.767 (0.056)	12.4	9.4	0.224	11.1	8.5	0.395
OS	31.1	21.4	0.014	0.724 (0.038)	0.776 (0.088)	31.1	21.9	0.187	28.2	21.0	0.460

Abbreviations: AE=adverse event; Gr=grade; HR=hazard ratio; mRCC=metastatic renal cell carcinoma; OS=overall survival; PFS=progression-free survival; SBP=systolic blood pressure.

a The multivariate analyses conducted for the individual AE models were similar to those conducted for the combined AE models; however, the covariates (i.e., prognostic factors) included in the full models differed as follows: the individual AE models included baseline vital characteristics (systolic blood pressure <140 *vs* ⩾140 mm Hg and diastolic blood pressure <90 *vs* ⩾90 mm Hg), whereas the combined AE models did not, and as outlined in Supplementary Tables 1 and 2, the combined AE models included prior nephrectomy, prior cytokine therapy, histology, baseline neutrophils, platelets, bone metastases, and any dose reduction during treatment, whereas the individual AE models did not; otherwise, all other covariates were the same in both models.

**Table 3:** Final combined AE multivariate models of associations between adverse events and objective response (OR; complete or partial response) for mRCC patients receiving sunitinib on Schedule 4/2 or any dose/schedule

		**Schedule 4/2**			Any dose/schedule		
Adverse event at any time point	End point	Odds ratio	95% CI	*P*-value^a^	Odds ratio	95% CI	*P*-value^a^
Neutropenia	OR	0.53	0.35–0.79	0.0021	0.44	0.30–0.66	<0.0001
Hypertension	OR	0.20	0.12–0.34	<0.0001	0.20	0.12–0.32	<0.0001
Hand–foot syndrome	OR	0.43	0.27–0.68	0.0003	0.43	0.28–0.66	0.0001
Asthenia/fatigue	OR	0.44	0.28–0.70	0.0005	0.48	0.32–0.73	0.0007
Thrombocytopenia	OR	0.54	0.33–0.87	0.0118	0.55	0.35–0.86	0.0098

Abbreviations: AE=adverse event; CI=confidence interval; mRCC=metastatic renal cell carcinoma.

a Two-sided Wald chi-squared test (all results were statistically significant).

**Table 4:** Final combined AE multivariate models of associations between adverse events and survival end points for mRCC patients receiving sunitinib on (A) Schedule 4/2 or (B) any dose/schedule

		Adverse event at any time point			Adverse event by the 12-week landmark		
Adverse event	End point	HR	95% CI	*P*-value^a^	HR	95% CI	*P*-value^a^
**(A) Schedule 4/2**							
Neutropenia	PFS	**0.77**	**0.61–0.97**	**0.0276**	**0.72**	**0.56–0.93**	**0.0130**
	OS	**0.65**	**0.50–0.85**	**0.0014**	**0.71**	**0.55–0.93**	**0.0122**
Hypertension	PFS	**0.37**	**0.27–0.52**	**<0.0001**	0.81	0.61–1.07	0.1305
	OS	**0.36**	**0.27–0.50**	**<0.0001**	**0.68**	**0.53–0.88**	**0.0036**
Hand–foot syndrome	PFS	0.90	0.70–1.15	0.3986	0.83	0.59–1.16	0.2651
	OS	**0.70**	**0.52–0.93**	**0.0152**	**0.64**	**0.44–0.94**	**0.0218**
Asthenia/fatigue	PFS	**0.56**	**0.42–0.74**	**<0.0001**	1.01	0.78–1.30	0.9555
	OS	0.82	0.61–1.10	0.1882	0.99	0.78–1.27	0.9586
Thrombocytopenia	PFS	0.83	0.63–1.10	0.1971	1.05	0.73–1.51	0.7905
	OS	0.96	0.70–1.33	0.8271	1.07	0.74–1.53	0.7233
**(B) Any dose/schedule**							
Neutropenia	PFS	**0.69**	**0.56–0.85**	**0.0004**	**0.72**	**0.57–0.91**	**0.0062**
	OS	**0.58**	**0.45–0.73**	**<0.0001**	**0.68**	**0.53–0.87**	**0.0019**
Hypertension	PFS	**0.44**	**0.33–0.58**	**<0.0001**	0.98	0.76–1.26	0.8730
	OS	**0.48**	**0.37–0.63**	**<0.0001**	**0.73**	**0.58–0.91**	**0.0063**
Hand–foot syndrome	PFS	0.88	0.70–1.10	0.2495	0.88	0.64–1.19	0.3963
	OS	**0.69**	**0.52–0.90**	**0.0062**	**0.60**	**0.42–0.86**	**0.0049**
Asthenia/fatigue	PFS	**0.69**	**0.54–0.88**	**0.0026**	0.98	0.79–1.23	0.8786
	OS	0.94	0.73–1.22	0.6576	0.96	0.77–1.19	0.7056
Thrombocytopenia	PFS	0.96	0.75–1.24	0.7557	1.09	0.79–1.51	0.5920
	OS	1.00	0.76–1.32	0.9863	1.11	0.81–1.52	0.5096

Abbreviations: AE=adverse event; CI=confidence interval; HR=hazard ratio; mRCC=metastatic renal cell carcinoma; OS=overall survival; PFS=progression-free survival.

Statistically significant results are in bold font.

a Two-sided Wald chi-squared test.
