# Supplementary material for: Sunitinib-associated hypertension and neutropenia as efficacy biomarkers in metastatic renal cell carcinoma patients
Source: Br J Cancer. 2015 Oct 22;113(11):1571–80. doi: 10.1038/bjc.2015.368 (PMC4705883; doi:10.1038/bjc.2015.368)
Supplement: Supplementary Tables [file bjc2015368x1.doc]

**Supplementary Table 1.** Final combined AE multivariate models of associations between all covariates and objective response (OR; complete or partial response) for mRCC patients receiving sunitinib on Schedule 4/2 or any dose/schedule

|  |  | **Schedule 4/2** | | |  | **Any dose/schedule** | | |
| --- | --- | --- | --- | --- | --- | --- | --- | --- |
| **Covariate** | **Endpoint** | **Odds ratio** | **95% CI** | ***P*-value^a^** |  | **Odds ratio** | **95% CI** | ***P*-value^a^** |
| Neutropenia | OR | 0.53 | 0.35–0.79 | **0.0021** |  | 0.44 | 0.30–0.66 | **<0.0001** |
| Hypertension | OR | 0.20 | 0.12–0.34 | **<0.0001** |  | 0.20 | 0.12–0.32 | **<0.0001** |
| Hand–foot syndrome | OR | 0.43 | 0.27–0.68 | **0.0003** |  | 0.43 | 0.28–0.66 | **0.0001** |
| Asthenia/fatigue | OR | 0.44 | 0.28–0.70 | **0.0005** |  | 0.48 | 0.32–0.73 | **0.0007** |
| Thrombocytopenia | OR | 0.54 | 0.33–0.87 | **0.0118** |  | 0.55 | 0.35–0.86 | **0.0098** |
| Age (<65 *vs* ≥65 years) | OR | 0.71 | 0.47–1.07 | 0.0982 |  | 0.67 | 0.46–0.99 | **0.0428** |
| Sex (male *vs* female) | OR | 0.79 | 0.49–1.26 | 0.3228 |  | 0.96 | 0.61–1.51 | 0.8719 |
| Ethnic origin  (White *vs* non-White) | OR | 0.70 | 0.32–1.51 | 0.3610 |  | 0.65 | 0.31–1.35 | 0.2434 |
| ECOG PS (0 *vs* 1/2) | OR | 0.72 | 0.47–1.09 | 0.1205 |  | 0.71 | 0.48–1.06 | 0.0909 |
| Time from diagnosis to treatment  (<1 *vs* ≥1 year) | OR | 0.76 | 0.51–1.14 | 0.1887 |  | 0.85 | 0.58–1.23 | 0.3811 |
| Prior nephrectomy  (yes *vs* no) | OR | 0.45 | 0.22–0.92 | **0.0293** |  | 0.52 | 0.27–1.02 | 0.0569 |
| Prior cytokine therapy  (yes *vs* no) | OR | 1.03 | 0.67–1.59 | 0.8963 |  | 1.40 | 0.95–2.06 | 0.0916 |
| Prior radiation therapy  (yes *vs* no) | OR | 1.23 | 0.72–2.11 | 0.4457 |  | 1.26 | 0.77–2.06 | 0.3552 |
| Histology (clear cell *vs* other) | OR | 0.94 | 0.52–1.69 | 0.8348 |  | 1.05 | 0.60–1.83 | 0.8693 |
| Sunitinib RDI (< *vs* ≥median) | OR | 1.51 | 1.00–2.29 | 0.0521 |  | 1.53 | 1.04–2.26 | **0.0320** |
| LDH (≥ *vs* <1.5 x ULN) | OR | 2.08 | 0.85–5.11 | 0.1089 |  | 1.84 | 0.75–4.49 | 0.1837 |
| Serum Hgb (≥ *vs* <LLN) | OR | 0.61 | 0.39–0.95 | **0.0278** |  | 0.59 | 0.39–0.90 | **0.0130** |
| Corrected serum calcium  (≥ *vs* <10.0 mg/dl) | OR | 0.80 | 0.43–1.48 | 0.4712 |  | 1.14 | 0.68–1.90 | 0.6135 |
| Baseline alkaline phosphatase  (≤ *vs* >ULN) | OR | 1.71 | 1.06–2.75 | **0.0268** |  | 1.62 | 1.04–2.50 | **0.0316** |
| BSA (> *vs* ≤1.82 m^2^) | OR | 0.84 | 0.51–1.39 | 0.5055 |  | 0.78 | 0.49–1.26 | 0.3137 |
| Baseline neutrophils  (> *vs* ≤ULN) | OR | 1.46 | 0.67–3.18 | 0.3364 |  | 1.25 | 0.64–2.45 | 0.5156 |
| Baseline platelets (> *vs* ≤ULN) | OR | 0.97 | 0.53–1.80 | 0.9325 |  | 1.00 | 0.58–1.74 | 0.9912 |
| Baseline bone metastasis  (yes *vs* no) | OR | 1.01 | 0.65–1.57 | 0.9650 |  | 1.17 | 0.78–1.75 | 0.4417 |
| Any dose reduction during treatment (yes *vs* no) | OR | 0.34 | 0.22–0.53 | **<0.0001** |  | 0.37 | 0.25–0.56 | **<0.0001** |

Statistically significant results in **bold font**.
**^a^**Two-sided Wald chi-squared test.
Abbreviations: BSA = body surface area; CI = confidence interval; ECOG PS = Eastern Cooperative Oncology Group performance status; Hgb = hemoglobin; LDH = lactate dehydrogenase; LLN = lower limit of normal; mRCC = metastatic renal cell carcinoma; OR = objective response; RDI = relative dose intensity; ULN = upper limit of normal.

**Supplementary Table 2.**  Final combined AE multivariate models of associations between all covariates and survival endpoints for mRCC patients receiving sunitinib on (A) Schedule 4/2 or (B) any dose/schedule

(A) Schedule 4/2

|  |  | **Adverse event  at any time point** | | |  | **Adverse event  by the 12-week landmark** | | |
| --- | --- | --- | --- | --- | --- | --- | --- | --- |
| **Covariate** | **Endpoint** | **HR** | **95% CI** | ***P*-value^a^** |  | **HR** | **95% CI** | ***P*-value^a^** |
| Neutropenia | PFS  OS | **0.77**  **0.65** | **0.61–0.97**  **0.50–0.85** | **0.0276**  **0.0014** |  | **0.72**  **0.71** | **0.56–0.93**  **0.55–0.93** | **0.0130**  **0.0122** |
| Hypertension | PFS  OS | **0.37**  **0.36** | **0.27–0.52**  **0.27–0.50** | **<0.0001**  **<0.0001** |  | 0.81  **0.68** | 0.61–1.07  **0.53–0.88** | 0.1305  **0.0036** |
| Hand–foot syndrome | PFS  OS | 0.90  **0.70** | 0.70–1.15  **0.52–0.93** | 0.3986  **0.0152** |  | 0.83  **0.64** | 0.59–1.16  **0.44–0.94** | 0.2651  **0.0218** |
| Asthenia/fatigue | PFS  OS | **0.56**  0.82 | **0.42–0.74**  0.61–1.10 | **<0.0001**  0.1882 |  | 1.01  0.99 | 0.78–1.30  0.78–1.27 | 0.9555  0.9586 |
| Thrombocytopenia | PFS  OS | 0.83  0.96 | 0.63–1.10  0.70–1.33 | 0.1971  0.8271 |  | 1.05  1.07 | 0.73–1.51  0.74–1.53 | 0.7905  0.7233 |
| Age (<65 *vs* ≥65 years) | PFS OS | 0.93 0.61 | 0.72–1.20 0.46–0.80 | 0.5634 **0.0005** |  | 1.04 0.71 | 0.79–1.36 0.54–0.93 | 0.7998 **0.0134** |
| Sex (male *vs* female) | PFS OS | 0.79 0.95 | 0.60–1.04 0.69–1.30 | 0.0870 0.7371 |  | 0.80 0.92 | 0.58–1.10 0.67–1.26 | 0.1620 0.6131 |
| Ethnic origin  (White *vs* non-White) | PFS OS | 0.85 0.87 | 0.55–1.31 0.54–1.40 | 0.4568 0.5701 |  | 0.69 0.90 | 0.42–1.12 0.56–1.45 | 0.1292 0.6749 |
| ECOG PS (0 *vs* 1/2) | PFS OS | 0.79 0.76 | 0.62–1.02 0.59–0.99 | 0.0672 **0.0398** |  | 0.89 0.72 | 0.67–1.18 0.55–0.93 | 0.4234 **0.0121** |
| Time from diagnosis to treatment  (<1 *vs* ≥1 year) | PFS OS | 1.16 1.48 | 0.92–1.46 1.15–1.91 | 0.2150 **0.0021** |  | 1.22 1.69 | 0.94–1.58 1.31–2.18 | 0.1264 **<0.0001** |
| Prior nephrectomy  (yes *vs* no) | PFS OS | 1.01 0.79 | 0.63–1.62 0.51–1.23 | 0.9759 0.2972 |  | 1.01 0.80 | 0.57–1.77 0.50–1.29 | 0.9817 0.3554 |
| Prior cytokine therapy  (yes *vs* no) | PFS OS | 1.28 1.67 | 1.00–1.64 1.26–2.21 | **0.0458 0.0003** |  | 1.25 1.75 | 0.95–1.64 1.33–2.32 | 0.1100 **<0.0001** |
| Prior radiation therapy  (yes *vs* no) | PFS OS | 1.10 1.02 | 0.80–1.52 0.73–1.44 | 0.5416 0.8855 |  | 1.16 1.07 | 0.81–1.67 0.75–1.52 | 0.4093 0.7132 |
| Histology (clear cell *vs* other) | PFS OS | 0.76 0.76 | 0.54–1.06 0.53–1.07 | 0.1088 0.1163 |  | 0.80 0.57 | 0.52–1.23 0.40–0.82 | 0.3063 **0.0021** |
| Sunitinib RDI (< *vs* ≥median) | PFS OS | 1.04 1.18 | 0.82–1.31 0.90–1.54 | 0.7776 0.2365 |  | 1.16 1.46 | 0.90–1.50 1.12–1.90 | 0.2637 **0.0057** |
| LDH (≥ *vs* <1.5 x ULN) | PFS OS | 1.60 1.86 | 0.91–2.82 1.07–3.24 | 0.1026 **0.0277** |  | 1.52 1.25 | 0.72–3.18 0.66–2.37 | 0.2685 0.4979 |
| Serum Hgb (≥ *vs* <LLN) | PFS OS | 0.82 0.67 | 0.63–1.07 0.51–0.87 | 0.1381 **0.0028** |  | 0.91 0.71 | 0.67–1.24 0.54–0.93 | 0.5539 **0.0142** |
| Corrected serum calcium  (≥ *vs* <10.0 mg/dl) | PFS OS | 0.86 1.51 | 0.60–1.24 1.06–2.16 | 0.4167 **0.0215** |  | 1.26 1.35 | 0.80–1.97 0.92–1.99 | 0.3178 0.1260 |
| Baseline alkaline phosphatase  (≤ *vs* >ULN) | PFS OS | 1.24 0.86 | 0.93–1.64 0.63–1.17 | 0.1395 0.3254 |  | 1.31 0.91 | 0.94–1.84 0.66–1.24 | 0.1102 0.5489 |
| BSA (> *vs* ≤1.82 m^2^) | PFS OS | 1.06 0.93 | 0.79–1.42 0.68–1.27 | 0.6901 0.6462 |  | 1.34 1.01 | 0.95–1.89 0.73–1.39 | 0.0970 0.9562 |
| Baseline neutrophils  (> *vs* ≤ULN) | PFS OS | 1.54 1.20 | 0.96–2.48 0.75–1.92 | 0.0724 0.4572 |  | 1.56 0.80 | 0.79–3.08 0.46–1.40 | 0.2022 0.4420 |
| Baseline platelets (> *vs* ≤ULN) | PFS OS | 1.88 1.64 | 1.29–2.75 1.12–2.39 | **0.0011 0.0102** |  | 2.36 1.83 | 1.44–3.88 1.22–2.74 | **0.0007 0.0036** |
| Baseline bone metastasis  (yes *vs* no) | PFS OS | 0.99  1.62 | 0.77–1.27 1.23–2.13 | 0.9139 **0.0006** |  | 1.24 1.55 | 0.93–1.64 1.17–2.06 | 0.1441 **0.0026** |
| Any dose reduction during treatment (yes *vs* no) | PFS OS | 0.55 0.58 | 0.43–0.70 0.44–0.76 | **<0.0001 0.0001** |  | 0.75 0.95 | 0.52–1.06 0.67–1.35 | 0.1002 0.7861 |

Statistically significant results in **bold font**.
^a^Two-sided Wald chi-squared test.
Abbreviations: BSA = body surface area; CI = confidence interval; ECOG PS = Eastern Cooperative Oncology Group performance status; Hgb = hemoglobin; HR = hazard ratio; LDH = lactate dehydrogenase; LLN = lower limit of normal; mRCC = metastatic renal cell carcinoma; OS = overall survival; PFS = progression-free survival; RDI = relative dose intensity; ULN = upper limit of normal.

(B) Any dose/schedule

|  |  | **Adverse event  at any time point** | | |  | **Adverse event  by the 12-week landmark** | | |
| --- | --- | --- | --- | --- | --- | --- | --- | --- |
| **Covariate** | **Endpoint** | **HR** | **95% CI** | ***P*-value^a^** |  | **HR** | **95% CI** | ***P*-value^a^** |
| Neutropenia | PFS  OS | **0.69 0.58** | **0.56–0.85 0.45–0.73** | **0.0004 <0.0001** |  | **0.72 0.68** | **0.57–0.91 0.53–0.87** | **0.0062 0.0019** |
| Hypertension | PFS  OS | **0.44 0.48** | **0.33–0.58  0.37–0.63** | **<0.0001**  **<0.0001** |  | 0.98 **0.73** | 0.76–1.26 **0.58–0.91** | 0.8730  **0.0063** |
| Hand–foot syndrome | PFS  OS | 0.88 **0.69** | 0.70–1.10  **0.52–0.90** | 0.2495 **0.0062** |  | 0.88 **0.60** | 0.64–1.19 **0.42–0.86** | 0.3963 **0.0049** |
| Asthenia/fatigue | PFS  OS | **0.69** 0.94 | **0.54–0.88** 0.73–1.22 | **0.0026** 0.6576 |  | 0.98 0.96 | 0.79–1.23 0.77–1.19 | 0.8786 0.7056 |
| Thrombocytopenia | PFS  OS | 0.96 1.00 | 0.75–1.24 0.76–1.32 | 0.7557 0.9863 |  | 1.09 1.11 | 0.79–1.51 0.81–1.52 | 0.5920 0.5096 |
| Age (<65 *vs* ≥65 years) | PFS OS | 0.96 0.73 | 0.77–1.20 0.58–0.93 | 0.7041 **0.0117** |  | 1.03 0.80 | 0.81–1.32 0.63–1.02 | 0.8146 0.0704 |
| Sex (male *vs* female) | PFS OS | 0.78 0.94 | 0.61–0.99 0.71–1.23 | **0.0437**  0.6361 |  | 0.87 1.01 | 0.65–1.16 0.76–1.34 | 0.3438 0.9572 |
| Ethnic origin  (White *vs* non-White) | PFS OS | 0.88 0.76 | 0.64–1.19 0.54–1.06 | 0.4066 0.1034 |  | 0.56 0.71 | 0.40–0.77 0.51–1.00 | **0.0005 0.0494** |
| ECOG PS (0 *vs* 1/2) | PFS OS | 0.77 0.72 | 0.62–0.95 0.57–0.90 | **0.0152 0.0035** |  | 0.89 0.72 | 0.70–1.13 0.57–0.91 | 0.3402 **0.0051** |
| Time from diagnosis to treatment  (<1 *vs* ≥1 year) | PFS OS | 1.32 1.45 | 1.07–1.62 1.15–1.83 | **0.0107 0.0015** |  | 1.50 1.72 | 1.19–1.90 1.36–2.17 | **0.0006 <0.0001** |
| Prior nephrectomy  (yes *vs* no) | PFS OS | 1.20 0.78 | 0.82–1.74 0.55–1.10 | 0.3479 0.1587 |  | 1.15 0.79 | 0.74–1.78 0.54–1.14 | 0.5425 0.2028 |
| Prior cytokine therapy  (yes *vs* no) | PFS OS | 1.30 1.46 | 1.05–1.61 1.15–1.84 | **0.0149 0.0016** |  | 1.33 1.63 | 1.04–1.69 1.28–2.08 | **0.0234 <0.0001** |
| Prior radiation therapy  (yes *vs* no) | PFS OS | 1.06 1.09 | 0.80–1.40 0.82–1.45 | 0.6806 0.5446 |  | 1.09 1.10 | 0.79–1.50 0.82–1.49 | 0.6208 0.5102 |
| Histology (clear cell *vs* other) | PFS OS | 0.80 0.72 | 0.58–1.10 0.52–0.99 | 0.1618 **0.0441** |  | 0.94 0.57 | 0.62–1.41 0.41–0.80 | 0.7502 **0.0011** |
| Sunitinib RDI (< *vs* ≥median) | PFS OS | 1.00 1.11 | 0.82–1.22 0.88–1.39 | 0.9637 0.3692 |  | 1.04 1.26 | 0.82–1.30 1.00–1.59 | 0.7671 0.0532 |
| LDH (≥ *vs* <1.5 x ULN) | PFS OS | 1.68 1.65 | 1.05–2.69 1.02–2.66 | **0.0313 0.0391** |  | 2.15 1.51 | 1.18–3.93 0.88–2.61 | **0.0124** 0.1390 |
| Serum Hgb (≥ *vs* <LLN) | PFS OS | 0.90 0.70 | 0.71–1.13 0.55–0.89 | 0.3571 **0.0039** |  | 0.98 0.75 | 0.75–1.28 0.59–0.96 | 0.8938 **0.0239** |
| Corrected serum calcium  (≥ *vs* <10.0 mg/dl) | PFS OS | 0.90 1.31 | 0.68–1.20 0.99–1.74 | 0.4659 0.0580 |  | 1.36 1.35 | 0.96–1.92 1.00–1.83 | 0.0814 0.0531 |
| Baseline alkaline phosphatase  (≤ *vs* >ULN) | PFS OS | 1.06 0.71 | 0.83–1.34 0.55–0.91 | 0.6545 **0.0072** |  | 1.19 0.76 | 0.90–1.57 0.59–0.99 | 0.2317 0.0436 |
| BSA (> *vs* ≤1.82 m^2^) | PFS OS | 1.12 1.08 | 0.88–1.43 0.82–1.41 | 0.3633 0.5925 |  | 1.24 1.04 | 0.94–1.64 0.79–1.37 | 0.1335 0.7756 |
| Baseline neutrophils  (> *vs* ≤ULN) | PFS OS | 1.21 1.14 | 0.85–1.74 0.80–1.64 | 0.2944 0.4547 |  | 1.35 1.04 | 0.83–2.19 0.70–1.57 | 0.2270 0.8341 |
| Baseline platelets (> *vs* ≤ULN) | PFS OS | 1.63 1.44 | 1.20–2.20 1.06–1.96 | **0.0016 0.0197** |  | 1.95 1.45 | 1.32–2.88 1.03–2.03 | **0.0007 0.0322** |
| Baseline bone metastasis  (yes *vs* no) | PFS OS | 1.09 1.49 | 0.87–1.37 1.17–1.89 | 0.4609 **0.0010** |  | 1.35 1.51 | 1.04–1.75 1.18–1.93 | **0.0222 0.0012** |
| Any dose reduction during treatment (yes *vs* no) | PFS OS | 0.56 0.58 | 0.46–0.70 0.46–0.74 | **<0.0001 <0.0001** |  | 0.90 1.08 | 0.67–1.20 0.81–1.44 | 0.4746 0.5885 |

Statistically significant results in **bold font**.
^a^Two-sided Wald chi-squared test.
BSA, body surface area; CI, confidence interval; ECOG PS, Eastern Cooperative Oncology Group performance status; Hgb, hemoglobin; HR, hazard ratio; LDH, lactate dehydrogenase; LLN, lower limit of normal; mRCC = metastatic renal cell carcinoma; OS, overall survival; PFS, progression-free survival; RDI, relative dose intensity; ULN, upper limit of normal.

**Supplementary Table 3.** Evaluation of eight different clinical scenarios (‘8-group’ analysis) on efficacy outcomes based on combined baseline neutrophil count and neutropenia grade and hypertension status during treatment for mRCC patients receiving sunitinib on (A) Schedule 4/2 or (B) any dose/schedule

(A) Schedule 4/2

|  |  | **Median time to progression/survival event, months (95% CI)** | |  |
| --- | --- | --- | --- | --- |
| **Group^a^** | ***n*** | **PFS** | **OS** | **OR, *n* (%)** |
| Baseline neutrophil count / neutropenia during treatment / hypertension during treatment | | | | |
| >ULN / nadir grade <2 / no HTN | 17 | 1.1 (1.0–2.5) | 4.1 (1.7–6.0) | 0 |
| >ULN / nadir grade ≥2 / no HTN | 0 | – | – | – |
| >ULN / nadir grade <2 / yes HTN | 16 | 8.1 (4.2–13.4) | 26.4 (11.4–31.0) | 7 (43.8) |
| >ULN / nadir grade ≥2 / yes HTN | 0 | – | – | – |
|  |  |  |  |  |
| ≤ULN / nadir grade <2 / no HTN | 50 | 2.8 (2.3–4.2) | 8.3 (5.3–13.1) | 2 (4.0) |
| ≤ULN / nadir grade ≥2 / no HTN | 25 | 3.9 (2.3–8.7) | 12.9 (7.2–23.1) | 6 (24.0) |
| ≤ULN / nadir grade <2 / yes HTN | 183 | 8.6 (7.9–10.7) | 20.3 (17.5–23.8) | 71 (38.8) |
| ≤ULN / nadir grade ≥2 / yes HTN | 243 | 16.1 (13.7–17.3) | 38.4 (34.3–45.4) | 159 (65.4) |

(B) Any dose/schedule

|  |  | **Median time to progression/survival event, months (95% CI)** | | |  |
| --- | --- | --- | --- | --- | --- |
| **Group^a^** | ***n*** | **PFS** | | **OS** | **OR, *n* (%)** |
| Baseline neutrophil count / neutropenia during treatment / hypertension during treatment | | | | | |
| >ULN / nadir grade <2 / no HTN | 34 | 1.7 (1.0–3.2) | 4.9 (2.6–7.0) | | 0 |
| >ULN / nadir grade ≥2 / no HTN | 2 | 12.6 (NA) | 13.5 (NA) | | 0 |
| >ULN / nadir grade <2 / yes HTN | 33 | 7.4 (4.2–8.6) | 24.5 (13.7–27.9) | | 9 (27.3) |
| >ULN / nadir grade ≥2 / yes HTN | 0 | – | – | | – |
|  |  |  |  | |  |
| ≤ULN / nadir grade <2 / no HTN | 68 | 2.7 (2.3–4.1) | 9.8 (5.7–12.3) | | 3 (4.4) |
| ≤ULN / nadir grade ≥2 / no HTN | 40 | 4.3 (2.6–8.7) | 16.8 (10.4–25.6) | | 7 (17.5) |
| ≤ULN / nadir grade <2 / yes HTN | 258 | 8.2 (7.8–10.2) | 19.1 (16.8–22.3) | | 74 (28.7) |
| ≤ULN / nadir grade ≥2 / yes HTN | 323 | 13.9 (13.4–16.3) | 37.1 (33.0–42.2) | | 172 (53.3) |

**^a^**Baseline neutrophil count was based on laboratory data, and neutropenia during treatment was based on adverse-event data, for which severity grade was based on neutrophil counts as well; hypertension was defined by SBP ≥140 mmHg.

Abbreviations: CI = confidence interval; HTN = hypertension; mRCC = metastatic renal cell carcinoma; NA = not available; OR = objective response; OS = overall survival; PFS = progression-free survival; SBP = systolic blood pressure; ULN = upper limit of normal.
